# Tryptophan-Rich *Moringa oleifera* Leaves Expand Plant Protein Potential: Nutritional Characteristics and Spectroscopic Fingerprinting

**DOI:** 10.3390/molecules31071188

**Published:** 2026-04-03

**Authors:** Joanna Harasym, Philippine Geollot, Gabriela Haraf, Rafał Wiśniewski, Adam Zając, Daniel Ociński, Ewa Pejcz

**Affiliations:** 1 Adaptive Food Systems Accelerator–Science Centre, Wroclaw University of Economics and Business, Komandorska 118/120, 53-345 Wroclaw, Poland; joanna.harasym@ue.wroc.pl (J.H.); rafal.wisniewski@ue.wroc.pl (R.W.); 2 Department of Biotechnology and Food Analysis, Wrocław University of Economics and Business, Komandorska 118/120, 53-345 Wroclaw, Poland; 3 ESIROI—Graduate School of Engineering Reunion Indian Ocean, University of La Réunion, 40 Avenue de Soweto, 97455 Saint-Pierre, France; philippine.geollot.pro@gmail.com; 4 Department of Food Technology and Nutrition, Wroclaw University of Economics and Business, 53-345 Wroclaw, Poland; gabriela.haraf@ue.wroc.pl; 5 Department of Bioorganic Chemistry, Wroclaw University of Economics and Business, Komandorska 118/120, 53-345 Wroclaw, Poland; adam.zajac@ue.wroc.pl; 6 Department of Chemical Technology, Wroclaw University of Economics and Business, 53-345 Wroclaw, Poland; daniel.ocinski@ue.wroc.pl

**Keywords:** *Moringa oleifera*, amino acid profile, protein quality, antioxidant activity, FT-IR spectroscopy, Raman spectroscopy, tryptophan

## Abstract

*Moringa oleifera* leaves are recognized as a nutrient-dense plant material of compositional and nutritional interest. This study aimed to characterize the nutritional and physicochemical properties of *M. oleifera* dried leaves through nutritional assessment and spectroscopic fingerprinting. Amino acid profiling, antioxidant activity assessment using ferric reducing antioxidant power (FRAP), 2,2-diphenyl-1-picrylhydrazyl (DPPH), 2,2′-azino-bis(3-ethylbenzothiazoline-6-sulfonic acid) (ABTS), and oxygen radical absorbance capacity (ORAC) assays, chromatographic analysis of organic acids and sugars, color measurement, techno-functional characterization, and vibrational spectroscopy including Fourier Transform infrared with attenuated total reflectance (FT-IR/ATR) and Raman were employed. The crude protein content was 16.13 ± 0.43%. Moringa leaves contained all essential amino acids, with notably high tryptophan content (amino acid score, AAS = 200.00%). The amino acids limiting the nutritional value of the protein were primarily sulfur-containing amino acids (AAS = 49.57%) and lysine (AAS = 49.79%). Histidine, leucine, and valine also showed levels below the reference protein. Antioxidant activity exhibited solvent-dependent patterns: the 80% ethanolic extract demonstrated significantly higher FRAP activity (27.05 ± 1.05 mg Trolox Equivalent (TxE)/g dry matter (DM)) and ORAC values (107.24 ± 6.80 mg TxE/g DM), while no statistically significant differences between extracts were observed for DPPH, ABTS, or total phenolic content. Chromatographic profiling identified fructose and glucose as the predominant sugars, alongside citric, succinic, lactic, and acetic acids. The leaves exhibited favorable techno-functional properties, including high water holding capacity and water solubility index. Spectroscopic analysis revealed bands consistent with proteins, lipids, carbohydrates, and glycoside-related structures, while the preserved green-yellow coloration (hue angle 101.68°) indicated retention of pigment-related features during processing. These findings provide compositional and physicochemical characteristics of Moringa leaves relevant to their evaluation as a plant-derived food material.

## 1. Introduction

*Moringa oleifera*, commonly known as the drumstick or horseradish tree, is a fast-growing, drought-resistant perennial native to the Indian subcontinent that thrives in tropical and subtropical climates globally [1]. Widely distributed across India, Africa, South America, and Southeast Asia, it is known by various vernacular names, including Sahjan, Sajna, Marango, Moringe, mulangay, and kelor, reflecting its cultural significance in traditional diets and medicinal practices [2]. Belonging to the family Moringaceae, this slender, softwood tree with feathery, compound leaves tolerates diverse environmental conditions, ranging from semi-arid to humid tropical zones, and grows on marginal soils, underscoring its agricultural potential [3].

The nutritional value of *Moringa oleifera* is related to its high protein content, essential minerals, vitamins, and bioactive compounds present in leaves, pods, roots, and seeds [4]. The leaves are particularly prized for their high nutrient density, which can substantially supplement the diets of populations vulnerable to malnutrition. Traditional medicine has long utilized various plant parts for their therapeutic properties, including for the treatment of diabetes, hypertension, inflammation, and infectious diseases. Pharmacological studies are increasingly validating these uses for drug discovery and the development of functional foods [5]. Additionally, Moringa offers economic and environmental benefits through sustainable cultivation, its use as a green manure and biopesticide, and its applications in animal feed and water purification [6]. Research continues to bridge knowledge gaps on nutrient and phytochemical bioaccessibility to optimize Moringa’s therapeutic potential [7], translating scientific evidence into practical frameworks for addressing malnutrition and chronic diseases globally, in line with recent Food and Agriculture Organization of the United Nations (FAO) reports highlighting *Moringa oleifera* as a nutrient-dense crop with potential applications in food security, sustainable diets, and community-level nutrition interventions in low- and middle-income regions [3,8].

Moringa leaves have a favorable macronutrient profile, containing approximately 20% protein with a relatively balanced essential amino acid composition compared with many plant sources [9]. The fat content remains relatively low, typically below 10%, and lipids are predominantly unsaturated fatty acids, especially oleic acid, which is beneficial for cardiovascular health [10]. Carbohydrates consist mainly of digestible polysaccharides, alongside appreciable dietary fiber, which support gastrointestinal health and metabolic parameters [11]. This balanced composition supports Moringa leaves’ role as a functional food ingredient contributing to energy, protein, and fiber requirements [12].

The leaves are distinguished by their high mineral content, including calcium, iron, potassium, and magnesium. Calcium supports bone health and muscular function, while iron combats iron-deficiency anemia, a prevalent global health concern [13]. Potassium and magnesium play crucial roles in cardiovascular regulation and enzymatic metabolism. Mineral bioaccessibility studies demonstrate a significant absorption potential due to the presence of low competing antinutrients and absorption-facilitating compounds [11]. Trace minerals, including zinc, manganese, and copper, contribute to enzymatic functions and antioxidant defenses, with concentrations varying by agroecological factors such as soil composition, climate, and cultivar genetics [6]. Modern techniques, including neutron activation analysis and atomic absorption spectroscopy, have provided nuanced insights into the nutritional value of Moringa and its suitability as a dietary supplement [14].

Moringa leaves are also an abundant source of bioactive phytochemicals. Phenolic compounds, including flavonoids and glucosinolates, contribute to antioxidant, anti-inflammatory, and antimicrobial properties [15]. Carotenoids, such as beta-carotene, vitamin C, and B-complex vitamins, contribute to the therapeutic portfolio. Specialized metabolites, including moringinine, quercetin, and chlorogenic acid, have been studied for their individual bioefficacies. Moringinine exhibits hepatoprotective and antiproliferative activities, while quercetin serves as a potent antioxidant that modulates cancer and inflammation signaling pathways [16]. Advanced analytical techniques such as LC-MS and molecular networking have revealed extensive flavonoid diversity, including glycosides and acetylated forms [17]. These phytochemicals represent critical functional ingredients underpinning Moringa’s nutraceutical and pharmaceutical potential.

The purpose of this study was to characterize selected nutritional and physicochemical properties of *Moringa oleifera* leaves using complementary analytical techniques. Specifically, the research aimed to evaluate the amino acid profile and amino acid score according to FAO/WHO reference patterns, to compare the antioxidant assay responses of water and ethanolic extracts, and to describe selected spectral features obtained by FT-IR/ATR and Raman analysis. The study was intended to provide compositional data on *Moringa oleifera* leaves rather than to establish functional or health-related effects.

## 2. Results and Discussion

### 2.1. Amino-Acid Characterization of M. oleifera Leaves

*Moringa oleifera* leaves are known as a rich source of protein. Crude protein content in *M. oleifera* dry leaves was noted as 16.13 ± 0.43%. The amino acids present at the highest concentrations in Moringa leaves are glutamic acid, aspartic acid, and leucine, whereas histidine, cysteine, and methionine are present at the lowest levels (Table 1). Similar relationships have been reported by other authors [18,19,20].

The biological value of protein is determined by the balance of essential amino acids (AA) [21]. Of the twenty amino acids that constitute proteins, eight are indispensable for humans and must be obtained through diet. According to the FAO Expert Consultation (FAO, 2013), protein quality can be assessed by comparing the composition of essential amino acids in a given protein to human requirements [13]. Moringa leaves contain all the essential amino acids (Table 1). However, the amino acids that limit the nutritional quality of Moringa protein are the sulfur-containing amino acids, with an amino acid score (AAS) of 49.57%. Lysine is the second-limiting amino acid, with an AAS of 49.79%. Compared to the reference protein, Moringa leaf protein also contains too little histidine (63.13%), valine (87.00%), and leucine (83.93%).

Notably, the tryptophan content in Moringa leaves protein is comparable to that of soybeans (1.3 g/100 g protein) and oilseeds such as rapeseed and sunflower (1.1–1.3 g/100 g protein) and higher than in legume proteins such as beans, broad beans and peas (0.0, 0.8, and 0.9 g/100 g protein, respectively) [22,23,24].

Tryptophan is an indispensable amino acid, and its inclusion allows a more complete assessment of the amino acid profile of the analyzed material. Elevated tryptophan levels are generally associated with protein-rich foods such as meat, fish, eggs, cheese, nuts, and oilseeds [25]. It should be emphasized that the digestibility of a protein determines the extent to which its amino acids can be utilized by the human body. Reported in vitro pepsin digestibility values for Moringa leaf protein vary widely, from 41.11% to 90.52% [20,26]. When corrected for digestibility, the amino acid score for sulfur-containing amino acids decreases to a range of 20.37–44.87%. Evidence also suggests that the digestibility of Moringa proteins can be enhanced by boiling in water [26].

To fully assess the nutritional value of a protein, the content of all indispensable amino acids, as well as cysteine and tyrosine, should be determined. A review of the available literature reveals a lack of studies reporting a complete profile of indispensable amino acids in *M. oleifera* leaf protein, which supports the relevance of including this parameter in the present study. Mune et al. [20], who analyzed *M. oleifera* leaf flour, reported higher levels of nearly all analyzed amino acids, except for cysteine, whose content was lower (0.41 g/100 g of protein). However, tryptophan was not determined in that study.

In other reports [18,19], the amino acid content of Moringa leaves (expressed as g/100 g of dry matter) was also generally higher, which may be partly attributed to a higher protein content (22.42% and 21.12%, respectively). In both of these studies, the contents of all amino acids required for a comprehensive evaluation of protein nutritional quality were not reported. The high tryptophan content reported by Shi et al. [18] is consistent with our findings.

### 2.2. Organic Acids and Simple Sugars Characterization of M. oleifera Leaves Powder

**Figure 1:**
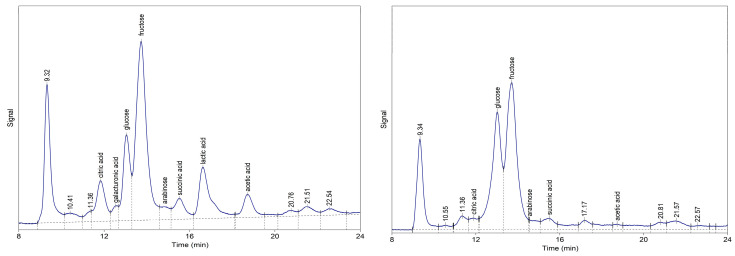
Chromatograms of water (**left**) and ethanolic (**right**) extracts of *M. oleifera* leaves.

The chromatographic profiles of water and ethanolic extracts of *M. oleifera* leaves (Figure 1, Table 2) revealed distinct patterns of organic acids and simple sugars, consistent with the known phytochemical composition of Moringa leaves.

Fructose was the predominant sugar in both extracts, with concentrations of 3.024 mg/mL and 2.569 mg/mL in water and ethanol extracts, respectively, followed by glucose (0.973 and 1.821 mg/mL). These findings align with previous reports indicating that glucose and fructose represent major soluble carbohydrate components in *M. oleifera* leaves [27]. The detection of arabinose in both extracts (0.255 and 0.166 mg/mL for water and ethanol, respectively) further confirms the diversity of monosaccharides present in Moringa leaf tissues.

Regarding organic acids, citric acid was identified in both extracts, with a higher concentration in the water extract (0.673 mg/mL) compared to ethanol (0.192 mg/mL). Succinic acid showed a similar pattern, with water extract yielding approximately twice the concentration (0.518 mg/mL) of the ethanolic extract (0.265 mg/mL). Notably, galacturonic acid (0.183 mg/mL) and lactic acid (1.275 mg/mL) were detected exclusively in water extracts, which is consistent with their higher polarity and stronger association with cell wall polysaccharides, particularly pectic substances [28]. The presence of tricarboxylic acid cycle intermediates such as citric and succinic acids reflects active primary metabolism in Moringa leaves, while lactic acid may indicate fermentation-related processes during sample preparation or natural leaf metabolism. Because compound assignment was based on retention time matching, these identifications should be treated as tentative.

### 2.3. Antioxidant Activity: Bioactive Compounds and Color of M. oleifera Leaves Powder

The antioxidant potential of *M. oleifera* leaf extracts demonstrated solvent-dependent variations across FRAP, DPPH, ABTS, and ORAC assays (Table 3).

The 80% ethanolic extract demonstrated significantly higher activity in the FRAP assay (27.05 mg TE/g DM) than the aqueous extract, maintaining statistical significance (*p* ≤ 0.05). In the remaining parameters, including DPPH (13.47–14.04 mg TE/g DM) and ABTS (2.59–2.67 mg TE/g DM), no statistically significant differences were observed between water and 80% ethanol. A similar trend was observed for total phenolic content (TPC), which reached approximately 19.5 mg GAE/g DM in both extracts. Particularly high values were obtained in the ORAC assay, amounting to 96.68 mg TE/g DM for the aqueous extract and 107.24 mg TE/g DM for the ethanolic extract. Notably, these ORAC values were substantially higher—up to several-fold—than those obtained from the remaining assays. This significant numerical disparity is a recognized characteristic of *M. oleifera* leaf extracts, consistently reported in the scientific literature [11,29,30].

This differential response pattern has been consistently reported in the literature and is attributed to the distinct antioxidant mechanisms captured by each assay and the differential solubility of bioactive compounds [31,32,33]. The presented results show that the aqueous extract exhibited the highest DPPH and ABTS radical scavenging activities, as well as the highest total phenolic content. Conversely, the ethanol extract showed superior reducing power and oxygen radical absorbance capacity. This suggests that water is more efficient at extracting phenolic compounds and specific radical scavengers, whereas ethanol extracts compounds with electron-transfer and broad-spectrum radical-quenching abilities, as assessed by FRAP and ORAC assays. The specific reaction mechanisms of each assay govern these differences. FRAP operates via single-electron transfer (SET), while ORAC is primarily a hydrogen atom transfer (HAT) based assay—a quality that best reflects in vivo physiological processes. DPPH and ABTS can involve both SET and HAT pathways [34,35]. The differences observed between assays and extraction media indicate that the measured values were dependent on both the analytical method applied and the extraction conditions.

The correlation between antioxidant activity and phenolic content in *M. oleifera* has been well-documented, with flavonoid compounds identified as key contributors to antioxidant activity. Bio-guided isolation studies have confirmed that specific glycosylated flavonoids, such as isoquercitrin and astragalin, along with phenolic acids, such as chlorogenic acid, are the predominant drivers of this high biological activity [11,29]. The higher DPPH and ABTS activities observed in the aqueous extract may be attributed to the efficient extraction of highly polar phenolic acids and ascorbic acid, which are effective radical scavengers. In contrast, the enhanced FRAP and ORAC activities in the ethanolic extract likely reflect the presence of moderately polar flavonoid glycosides and aglycones, such as quercetin and kaempferol derivatives, which are potent reductants [36,37].

**Figure 2:**
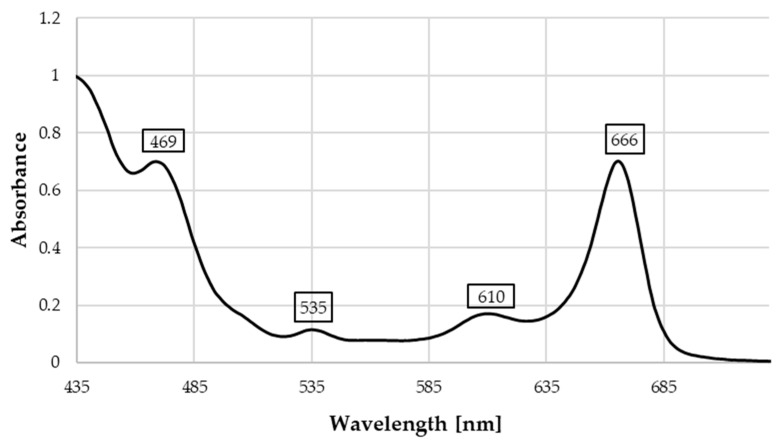
VIS spectrum of methanol extracts of *M. oleifera* leaves. (4 mg/mL).

Furthermore, the analysis of photosynthetic pigments revealed a significant predominance of chlorophyll a (2.12 mg/g DM) over chlorophyll b (0.37 mg/g DM), with carotenoids present at 0.61 mg/g DM. These results are consistent with literature values for carotenoid concentrations in Moringa leaves, which are significantly higher than those of other common vegetables such as spinach [35]. The total antioxidant potential is further enhanced by the presence of these stable pigments and carotenoids, which synergize with polyphenols to increase the total biological potential of the leaves [38,39]. The phytochemical integrity and successful preservation of bioactive components during processing are confirmed by spectral analysis, which identified characteristic absorption maxima at 666 nm (chlorophyll a) and 469 nm (carotenoids) (Figure 2). Additional peaks at 535 nm and 610 nm are typical of chlorophyll spectra and may result from the presence of stable pigment derivatives, such as pheophytins. The formation of pheophytins, caused by the replacement of the central magnesium ion with hydrogen, is a common phenomenon during drying and processing and contributes to the stability of the final extract. The constant positioning of these peaks and the absence of significant “red shifts” indicate that the bioactive profile was effectively preserved, making these extracts suitable for food applications.

The color characteristics of dried *M. oleifera* leaves in the CIELab system showed moderate lightness (L* = 48.57 ± 0.42), negative a* values (−3.56 ± 0.04) indicating green hue, and positive b* values (14.53 ± 0.10) reflecting yellow tones. The calculated chroma (21.39 ± 0.08) and hue angle (101.68 ± 0.05°) confirm a characteristic green-yellow coloration typical of dried leafy materials.

The techno-functional properties of *Moringa oleifera* dry leaves revealed favorable water-binding characteristics, with a water holding capacity (WHC) of 9.47 ± 1.38 and water absorption capacity (WAC) of 4.50 ± 0.11 g water/g DM. Oil absorption capacity (OAC) reached 2.65 ± 0.05 g oil/g dry mass, yielding a hydrophilic/lipophilic index (HLI) of 1.70 ± 0.04, indicating a predominantly hydrophilic nature. The water absorption index (WAI) and swelling power (SP) were 5.63 ± 0.36 g water/g DM and 7.15 ± 0.60 g water/g DM, respectively, while the water solubility index (WSI) was notably high at 21.24 ± 1.94 g/100 g DM, suggesting substantial soluble component content. Regarding surface-active properties, the leaves exhibited considerable foaming capacity (67.50 ± 2.65 mL) with good foam stability (77.11 ± 2.60%), whereas emulsifying activity (6.04 ± 0.55%) and emulsion stability (8.66 ± 0.57%) were comparatively modest.

These values are comparable to those reported for gently dried Moringa leaves in previous studies, where L* values around 50 and hue angles exceeding 100° have been documented [28,38]. The moderate lightness and preserved green-yellow hue indicate good retention of chlorophylls during the drying process, which is significant given that chlorophyll content correlates positively with antioxidant capacity [40,41,42]. The negative a* value confirms the predominance of green pigmentation over red, while the hue angle positioning in the second quadrant of the color space is characteristic of chlorophyll-rich plant materials. These color parameters suggest that the drying conditions employed preserved bioactive pigments, which may contribute to the observed antioxidant activities [43].

### 2.4. Mid-Infrared and Raman Spectra Characteristics of M. oleifera Leaves Powder

**Figure 3:**
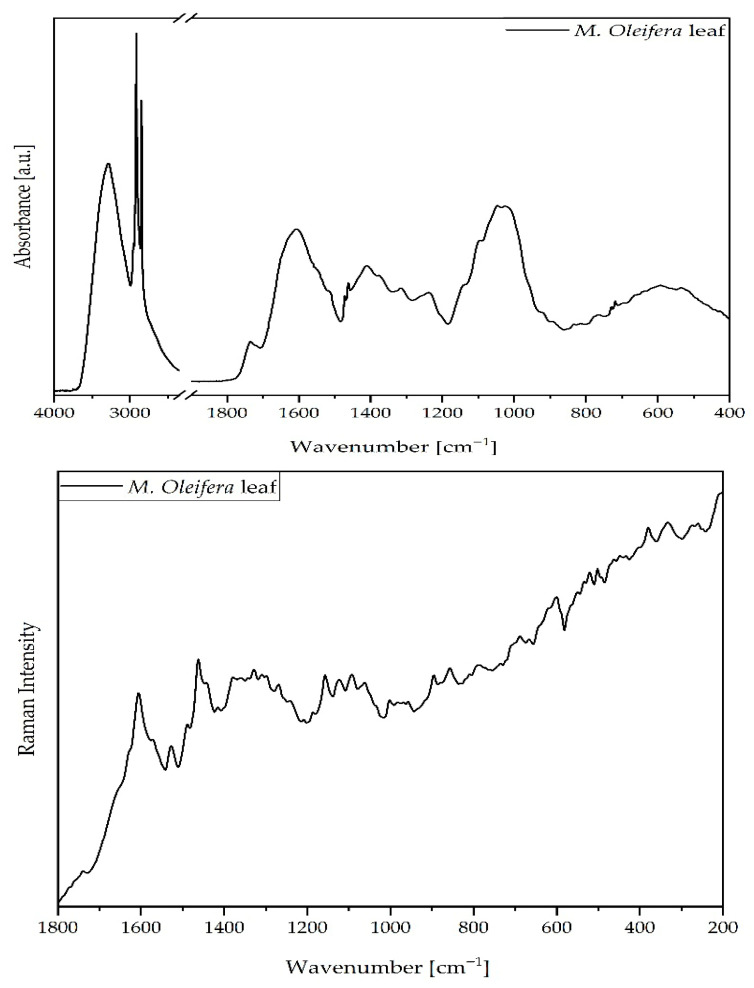
FT-IR/ATR (**top**) and FT-Raman (**bottom**) spectra of *M. oleifera* leaves.

The FT-IR/ATR and Raman spectra of *Moringa oleifera* leaves (Figure 3) revealed a wide distribution of vibrational bands, reflecting the presence of proteins, lipids, polysaccharides, and a broad range of phenolic compounds.

Detailed assignments of the observed signals are presented in Table 4. The spectra highlight the complex phytochemical composition of the leaves, with a particularly high contribution from proteinaceous structures and lipid-derived metabolites, consistent with the plant’s nutritional profile.

The strong and broad band at 3288 cm^−1^ was attributed to O–H stretching vibrations, mainly from hydroxyl groups in, e.g., carbohydrates, flavonols, and proteins. This feature is typical of plant-based matrices rich in polysaccharides and phenolic hydroxyl groups [31,51]. The intense band at 2915 and 2849 cm^−1^, corresponding to asymmetric and symmetric CH_2_ stretching, is consistent with the presence of lipid-related structures. The presence of lipids was further supported by the carbonyl stretching bands at 1736 cm^−1^ and 1724 cm^−1^, assigned to esters of fatty acids, flavonoids, and other lipid derivatives (Figure 2 and Table 4). These results demonstrate the abundance of bioactive lipidic compounds in *M. oleifera* leaves, which have been previously associated with cardioprotective and anti-inflammatory activities [52,53,54,55].

In the amide region, several distinct bands confirm the high protein content of the leaves. The band at 1650 cm^−1^ (Amide I) originates from C=O stretching vibrations of the peptide backbone, while the band at 1550 cm^−1^ (Amide II) is associated with N–H bending and C–N stretching. The overlap at 1607 cm^−1^ includes contributions from aromatic C=C stretching in flavonoids and phenolic compounds and N–H vibrations from proteins (Figure 3 and Table 4). These results support previous compositional analyses reporting that *M. oleifera* leaves contain *c.a.* 22–30% protein in dry weight, with an essential amino acid profile suitable for nutritional supplementation [10,51]. The deformation vibrations at 1472 and 1463 cm^−1^, arising from CH_2_ and CH_3_ groups in proteins and lipids, and the symmetric CH_3_ deformation at 1377 cm^−1^ confirm protein abundance.

The aromatic region (1519–1607 cm^−1^) also revealed strong contributions from flavonoid and lignin structures. The band at 1519 cm^−1^ corresponds to aromatic C=C stretching in flavonoids and lignins, while the shoulder at 1550 cm^−1^ suggests overlapping proteinaceous contributions. Furthermore, the band at 1411 cm^−1^, assigned to O–H deformation in phenols and carboxylic acids (Figure 3 and Table 4), highlights the presence of polyphenolic compounds and supports the role of the leaves as a significant source of antioxidant metabolites [56].

The absorption at 1238 cm^−1^ was attributed to in-plane bending C–O–H vibrations of esters and phospholipids, while the shoulder at 1204 cm^−1^ corresponds to C–O stretching in esters and flavonoids. Bands observed between ca. 1186 and 895 cm^−1^ confirmed the presence of polysaccharides and glycosides. The distinct band at 1138 cm^−1^ is particularly important, assigned to C–O–C stretching vibrations in glycosides (Figure 3 and Table 4). This feature has been reported in the literature for plant matrices containing glycosidic and carbohydrate-related structures [57]. In the present study, it is discussed only as a descriptive band assignment.

The prominent aromatic C=C stretching vibrations observed at 1605–1607 cm^−1^ in both FT-IR and Raman spectra provide direct spectroscopic evidence for the presence of flavonoids and phenolic compounds, which have been identified as key contributors to the antioxidant attributes of *M. oleifera* extracts, demonstrating strong correlations with FRAP (r = 0.844) and DPPH (r = 0.947) assays [33,57]. Furthermore, the broad O–H stretching band at 3288 cm^−1^, attributed to hydroxyl groups in flavonols and phenolic compounds, reflects the abundance of hydrogen-donating functional groups that are directly responsible for the high radical scavenging activity observed in antioxidant assays [34,51]. The preserved green-yellow coloration indicated by the hue angle (101.68°) correlates with the spectroscopic detection of chlorophyll-associated bands and carotenoid signatures, as chlorophyll retention during drying is known to positively influence both visual quality and antioxidant capacity of *M. oleifera* leaves [30,40].

Additional carbohydrate-associated signals were observed at 1097, 1046, and 1024 cm^−1^, corresponding to C–O stretching in carbohydrates, secondary alcohols, and cellulose. The peaks at 922 and 895 cm^−1^ were linked to pyranoid ring vibrations and aromatic C–H out-of-plane bending, while the bands at 834, 770, 729, and 718 cm^−1^ reflected aromatic and alkyl C–H bending modes. Finally, skeletal and ring deformation vibrations at 594 and 533 cm^−1^ were attributed to COO^−^ groups, further supporting the presence of carboxylated compounds and phenolic acids (Figure 3 and Table 4).

These results align with previous reports emphasizing the leaves’ nutritional and therapeutic value, particularly in preventing malnutrition and developing functional foods and nutraceuticals [58,59].

## 3. Materials and Methods

### 3.1. Leaves Harvesting and Preparation

Leaves and seeds of *Moringa oleifera* have been harvested in the city of Saint-Paul, Reunion Island, France, around the following locations: 21°03′10.9″ S 55°13′45.9″ E; 21°03′44.1″ S 55°13′43.1″ E and 21°05′40.7″ S 55°14′32.9″ E. The leaves were blanched and dried at 50 °C for 1 h to preserve the compounds. Then, they were vacuum-packed and stored in a cold chamber at 4 °C until analysis.

### 3.2. The Crude Protein Content

The nitrogen content of each flour and blend was analyzed using the Kjeldahl method, and protein content was estimated using a nitrogen-to-protein conversion factor of 6.25 (AOAC method 954.01) [60]. A total of 0.5 g of ground sample was added to 20 cm^3^ of concentrated sulfuric acid and 2 Kjeldahl tablets (containing 3.5 g K_2_SO_4_ and 0.4 g CuSO_4_·5H_2_O) for sample mineralization. Distillation was performed using KjelFlex K-360 (BÜCHI Labortechnik GmbH, Essen, Germany), following the manufacturer’s procedure. Titration was conducted by adding approximately 5 drops of Toshiro indicator to the conical flask placed in the distiller, followed by titration with 0.1 M HCl until the color changed or pH reached approximately 4.6. The nitrogen content in the sample N (%) and protein content Y (%) were calculated according to Equations (1) and (2):

N = ((a + b) × n × M(N) × F)/(m × 1000) × 100%.(1)

%Protein = %N × 6.25(2)

where a = volume of standard HCl used for sample titration (cm^3^); b = volume of standard HCl used for titration of the blank sample (cm^3^); n-molarity of HCl; M(N) = atomic mass of nitrogen (14.007 g/mol); F = molar reaction coefficient (for HCl = 1); m = mass of the tested sample (g); 1000-conversion factor (change unit from cm^3^ to dm^3^).

### 3.3. Determination of Amino Acids (AA) Content

Amino acid content was determined by acid hydrolysis, oxidation followed by acid hydrolysis (methionine and cysteine), and alkaline hydrolysis (tryptophan). Briefly, for amino acid determination by acid hydrolysis, 0.1 g (with an accuracy of 0.0001 g) of powdered Moringa leaves was hydrolyzed at 110 °C for 24 h in tightly sealed vials under a nitrogen atmosphere using 3 mL of 6 M HCl with 0.15 mL of a 5% phenol solution. After hydrolysis, the cooled sample was filtered through Whatman No. 1 filter paper into a flask, and the vial and filter were rinsed twice with HPLC-grade H_2_O. At this stage, 0.5 mL of a 1 nM sarcosine solution was added to the sample as an internal standard. The flask was then connected to a vacuum evaporator, and the filtrate was evaporated to dryness at 65 °C. The dry hydrolysate was rinsed with 3 mL of HPLC-grade water and evaporated again. The dry residue was transferred with 0.1 M HCl into a 5 mL volumetric flask and made up to volume. The prepared sample was filtered through a 0.22 μm syringe filter and subjected to HPLC analysis.

Sulfur-containing amino acids (methionine and cysteine) were determined after performic acid oxidation, which converts them to cysteic acid and methionine sulfone, respectively, before acid hydrolysis with 6 M HCl. The resulting compounds were subsequently recalculated to their corresponding amino acids. The oxidation mixture was prepared by mixing hydrogen peroxide and formic acid at a 1:9 ratio. The mixture was left at room temperature for 1 h with occasional mixing, then cooled in a refrigerator for 30 min. The sample (0.25 g ± 0.0001 g) was placed in a flask, treated with 8 mL of performic acid, and left overnight in a refrigerator. The following day, the oxidation mixture was evaporated to dryness under vacuum at 50 °C. The dry residue was then rinsed with 3 mL of HPLC-grade water and evaporated again. The evaporated sample was treated with 3 mL of 6 M HCl, transferred to a vial, purged with nitrogen, and tightly sealed with a rubber stopper and an aluminum cap. Acid hydrolysis was then carried out as described above.

Tryptophan was determined using the alkaline hydrolysis method described by Çevikkalp et al. 2016 [61]. In a 20 mL vial, 0.50 g (±0.0001 g) of powdered Moringa leaves was weighed and mixed with 10 mL of 5 M NaOH (de-aerated by bubbling with N_2_ for 10 min). The vials containing the samples were purged with nitrogen (for 30 s) and immediately sealed with a rubber stopper and crimped with an aluminum cap. Hydrolysis was carried out at 110 °C for 12 h. After hydrolysis and cooling of the samples, the hydrolysate was filtered through Whatman No. 1 filter paper. Then, 1 mL of the filtrate was transferred to a small beaker, and 20 mL of HPLC-grade water was added. The solution was adjusted to pH 6.3 using HCl. Subsequently, the sample was transferred to a 50 mL volumetric flask and made up to volume with HPLC-grade water. The solution, after filtration through a 0.22 μm syringe filter, was ready for chromatographic analysis.

Amino acid analysis was performed using an Agilent 1100 Series HPLC system (Agilent Technologies, Santa Clara, CA, USA) equipped with an autosampler and two detectors: UV-DAD and FLD. Amino acids were quantified by comparison with external amino acid standards. L-aspartic acid, glycine, L-alanine, L-valine, L-leucine, L-isoleucine, L-serine, L-threonine, L-tyrosine, L-proline, L-arginine, L-histidine, L-glutamic acid, L-phenylalanine, and L-lysine were purchased from Agilent Technologies (Santa Clara, CA, USA) as standard solutions in 0.1 M HCl at concentrations of 100 pmol/mL, 250 pmol/mL, and 1 nmol/mL. L-tryptophan and sarcosine (internal standard) were obtained in powder form (Amino Acid Supplement Kit, Agilent Technologies, Santa Clara, CA, USA). For the analysis of cysteine and methionine, L-cysteic acid monohydrate (≥99%; Acros Organics, Thermo Fisher Scientific, Waltham, MA, USA) and methionine sulfone (≥98%; Sigma-Aldrich, Merck, Darmstadt, Germany) were used as standards. All reagents used were of HPLC grade.

The separation of amino acids from samples after acid hydrolysis was performed according to the method of Henderson et al. 2000 [62] using a UV-DAD detector and an AAA Eclipse Zorbax column with a length of 150 mm, a diameter of 3 mm, and porosity of 3.5 μm (Agilent Technology Inc., Santa Clara, CA, USA). Automated, online derivatization using o-phthalaldehyde (OPA) for primary amino acids and 9-fluorenylmethyl chloroformate (FMOC) for secondary amino acids were used. The reagents needed for derivatization: OPA, FMOC, as well as Borate Buffer (0.4 M in water; pH 10.2) were purchased from Agilent Technology Inc. (St. Clara, CA, USA). The detection parameters were 338 nm for OPA-amino acids and 262 nm for FMOC-amino acids. The temperature was controlled at 40 °C. The mobile phase was 40 mM Na_2_HPO_4_, adjusted to pH 7.8 with a 10.0 M NaOH solution (solvent A) and water:acetonitrile:methanol (45:45:10 *v*/*v*/*v*, solvent B) at the flow rate 2 mL/min with gradient elution program for 26 min (initially 0% B; increased linearly to 57% B from 1.9 to 18.1 min; then increased linearly to 100% B at 18.6 min and held for 4.7 min; subsequently decreased linearly to 0% B at 23.2 min and held for 2.8 min—stop-time after 26 min). The calibration curve was constructed using three standard solutions with concentrations of 90, 225, and 900 pmol/μL. Validation parameters of the method are presented in Table 5.

HPLC analysis of sulfur-containing amino acids (cysteine and methionine) was performed according to Varzaru et al. 2013 [63] using a Hypersil BDS C18 column (250 × 4.6 mm, 5 μm particle size; Thermo Fisher Scientific, Waltham, MA, USA). Automated online derivatization with OPA, FMOC, and borate buffer (pH 10.2) was applied [62] using reagents supplied by Agilent Technologies (Santa Clara, CA, USA). The column temperature was set at 45 °C. The mobile phase consisted of solvent A (28 mM Na_2_HPO_4_, pH 7.8) and solvent B (water:acetonitrile:methanol, 20:20:60; *v*/*v*/*v*). The flow rate was 1.7 mL/min. The gradient elution program was as follows: 0–2 min, 0% B; 2–25 min, linear increase to 57% B; 25–26 min, linear increase to 100% B; 26–29 min, 100% B; 29–30 min, linear decrease to 0% B; 30–35 min, 0% B. The total run time was 35 min. The calibration curve was constructed using five standard solutions of cysteic acid and methionine sulfone at concentrations ranging from 25 to 200 μg/mL. Validation parameters for cysteic acid were as follows: LOD 1.39 μg/mL, LOQ 10.82 μg/mL, RSD 0.70%, and R^2^ 0.99961. For methionine sulfone, the following values were obtained: LOD 3.57 μg/mL, LOQ 10.82 μg/mL, RSD 2.74%, and R^2^ 0.99970.

Tryptophan was quantified according to the procedure described by Çevikkalp et al. 2016 [61] using a Lichrospher 60 RP-Select B column (250 × 4 mm, particle size 5 μm) purchased from Agilent Technologies (Santa Clara, CA, USA). The mobile phase was prepared by mixing 900 mL of ammonium acetate buffer (pH 6.3) with 100 mL of acetonitrile. The flow rate was set at 1 mL/min, and the injection volume was 10 μL. TRP was detected using an FLD detector at 280 nm excitation and 520 nm emission. The run time was 10 min. The calibration curve was constructed using 4 standard solutions with concentrations ranging from 2.5 to 15 μg/mL. Validation parameters for tryptophan were as follows: LOD 0.02 μg/mL, LOQ 0.06 μg/mL, RSD 1.67%, and R^2^ 0.99998. Method accuracy was evaluated using a matrix spike recovery test at two fortification levels (5 and 15 μg/mL), yielding recoveries of 97.6–98.3% [64].

Amino acid score (AAS) calculation:

AAS = ((g of AA in 100 g of a protein of Moringa leaf)/(g of AA in 100 g of requirement pattern)) × 100%

The AA with the lowest AAS value is called the limiting amino acid. Values for AAS were calculated using the recommended requirement patterns from FAO/WHO [41]. The reference scoring pattern (g/100 g protein) for adults was: His 1.6; Ile 3.0; Leu 6.1; Lys 4.8; sulfur AA 2.3; aromatic AA 4.1; Thr 2.5; Trp 0.66; Val 4.0.

### 3.4. Extraction of Bioactive Compounds

The extraction of bioactive compounds from the blends was performed as described in ref. [65], with slight modifications. Specifically, 0.5 g of each sample was mixed with 10 mL of water and 80% ethanol in water, and homogenized for 1 min using a vortex mixer (MX-S, Chemland, Stargard, Poland). The homogenized mixtures were then agitated on a laboratory rotary shaker (MX-RD PRO, Chemland, Stargard, Poland) at room temperature for 2 h to maximize compound extraction. After shaking, the samples were centrifuged at 3500× *g* for 10 min at 4 °C (MPW-350, MPW MED. INSTRUMENTS, Warsaw, Poland). To determine total chlorophylls (TChl) and carotenoids (TC), an additional extraction was performed under the same conditions using pure methanol, and the agitation step was conducted in darkness to prevent pigment degradation. The clear supernatants obtained were stored at 4 °C until further spectrophotometric analysis.

### 3.5. Organic Acids and Simple Sugars Chromatographic Analysis

Chromatographic analysis was performed using an HPLC system equipped with a pump (Knauer Smartline 1000, Berlin, Germany), vacuum degasser (Knauer Smartline Manager 5000, Berlin, Germany), autosampler (Knauer Azura AS 6.1 L, Berlin, Germany), column thermostat (Knauer Jetstream 2 Plus, Berlin, Germany), and a refractive index detector (Knauer RI detector K–2300, Berlin, Germany). Separation of organic acids and saccharides was carried out using the column Shodex SUGAR SH1011 8.0 × 300 mm with a guard column Shodex SUGAR SH-G 6.0 × 50 mm (Tokyo, Japan); eluent: 5 mM H2SO4, temperature: 60 °C, flow rate: 0.6 mL/min. Software Clarity Chrome^®^ v. 9.1 was the data handling system. Retention time matching was used to identify the peaks.

### 3.6. DPPH Radical Scavenging Activity

A total of 0.0345 mL of sample was mixed with 1 mL of a 0.1 mM methanolic DPPH solution. After 20 min, the absorbance was measured at 517 nm. The antioxidant capacity was then expressed as milligrams of Trolox equivalent (TE) per gram of sample dry matter (DM).

### 3.7. ABTS Reduction

A total of 0.0204 mL of each sample was mixed with 1.0 mL of a diluted ABTS+ solution prepared as described in ref. [65]. The absorbance of the mixture was measured at 734 nm, 10 s after combining the sample with the ABTS+ solution. A calibration curve was constructed using Trolox as the standard, with concentrations ranging from 100 to 800 μmol/L. The antiradical capacity was expressed as milligrams of Trolox equivalent (TE) per gram of sample DM.

### 3.8. FRAP Assay

A total of 0.0345 mL of each sample was mixed with 0.998 mL of a freshly prepared FRAP solution, as outlined elsewhere [65]. Following a 15 min incubation, the absorbance of the mixture was measured at 593 nm. The results were expressed as milligrams of TE per gram of the sample DM.

### 3.9. Determination of Total Polyphenols

Total polyphenolic content (TPC) was determined using the Folin–Ciocalteu method, as described in ref. [66], with slight modifications. Briefly, 0.02 mL of each sample was mixed with 1.58 mL of distilled water, followed by the addition of 0.1 mL of Folin–Ciocalteu reagent. After 5 min, 0.3 mL of 20% sodium carbonate (Na_2_CO_3_) solution was added, and the mixture was incubated in the dark at 38 °C for 30 min. The absorbance was measured at 765 nm using a spectrophotometer. Gallic acid was used as the calibration standard, and the results were expressed as milligrams of gallic acid equivalents (GAE) per gram of powder dry matter.

### 3.10. ORAC Assay

The oxygen radical absorbance capacity (ORAC–FL) assay was performed according to the method described by Ou et al. [66], with slight modifications. Fresh fluorescein solution (80 nmol, 150 μL) was mixed with 25 μL of sample, standard, or phosphate buffer (75 mmol, pH 7.4) in a black 96-well microplate and incubated at 37 °C for 15 min. The reaction was then initiated by adding 25 μL of AAPH (153 nmol). Fluorescence intensity was measured using a FLUOstar Omega microplate reader (BMG Labtechnologies GmbH, Offenburg, Germany) with a top optic, at excitation and emission wavelengths of 485 nm and 520 nm, respectively. Phosphate buffer (75 mmol, pH 7.4) was used as a blank. Measurements were recorded every 150 s over a total analysis time of 100 min. Trolox standard solutions (1.6–25 µg/mL) were used for calibration, and the results were expressed as mg Trolox equivalents (TE) per gram of dry matter.

### 3.11. Determination of Chlorophylls (TChl) and Carotenoids (TC)

Absorbance of methanol extracts was measured at 470 nm, 652.4 nm, and 665.2 nm, and a full visible (VIS) spectrum scan was recorded. TChl (as the sum of chlorophyll a and b content) and TC were calculated using the equations of Wellburn [67] for methanol and high-resolution spectrophotometers. The results were converted to milligrams per gram of powder DM.

### 3.12. Techno-Functional Features

The functional properties examined included moisture content, water-holding capacity (WHC), water-absorption capacity (WAC), water-absorption index (WAI), swelling power (SP), water-solubility index (WSI), oil-absorption capacity (OAC), and hydrophilic/lipophilic index (HLI), which was measured as described in ref. [68]. Moisture determination followed the gravimetric approach outlined in AACC Method 44-19.01, with samples dried at 135 °C using a laboratory oven (SML, Zalmed, Łomianki, Poland).

For WHC and WAC measurements, 3 g portions were suspended in 30 mL of distilled water, vortexed, and centrifuged at 3000× *g*. The mass of water retained in the pellet after supernatant removal was used to express WAC as grams of absorbed water per gram of dry matter.

WAI, WSI, and SP were determined by dispersing 3 g of sample in 30 mL of distilled water, heating at 90 °C for 10 min in a thermostatically controlled water bath (MLL147, AJL Electronics, Kraków, Poland), and then cooling. After centrifugation (3000× *g*, 10 min), the sediment weight provided WAI values. The supernatant fraction was dried at 110 °C for 24 h to quantify soluble components, enabling calculation of WSI (expressed per 100 g dry matter) and SP (grams of water retained per gram of insoluble dry residue).

OAC was assessed by combining 3 g of sample with 30 mL rapeseed oil, vortexing for 30 s, and centrifuging at 3000× *g*. Following supernatant removal, residual oil was evaporated at 50 °C (Vindon Scientific, Loughborough, UK), and OAC was expressed as grams of oil absorbed per gram of dry sample. The HLI was subsequently derived from the WAC/OAC ratio.

Foaming capacity (FC) and foam stability (FS) were assessed by dispersing 1 g of sample in 50 mL of distilled water in a graduated cylinder, then vigorously agitating for 5 min. Volume measurements before and after agitation enabled FC calculation (mL/g dry matter). FS was determined by recording the foam volume remaining after a 60 min standing period, expressed as a percentage of the initial foam volume.

Emulsifying activity (EA) and emulsion stability (ES) were evaluated by combining the sample with water and oil, homogenizing the mixture, and measuring the emulsion layer volume following centrifugation. ES was further assessed by subjecting the emulsion to thermal treatment and monitoring phase separation behavior.

Chromatic attributes were measured using a CR-310 colorimeter (Konica Minolta, Ramsey, NJ, USA) fitted with a CR-A50 granular sample holder. The instrument was calibrated against standard black-and-white reference tiles prior to each measurement session. The CIE L*a*b* color coordinates—representing lightness (L*), the green–red axis (a*), and the blue–yellow axis (b*)—were obtained as the mean of four replicate measurements per sample.

### 3.13. Fourier Transform Infrared Spectroscopy (FTIR)

The FTIR spectra of the samples were recorded using a Nicolet 6700 FT-IR spectrophotometer (Thermo Fisher Scientific, MA, USA) equipped with a diamond crystal cell for attenuated total reflection (ATR) mode. Each sample was scanned 64 times within the wavelength range of 4000–500 cm^−1^ at a resolution of 4 cm^−1^. To ensure precise measurements, background correction was applied using the air spectrum.

### 3.14. Statistical Analysis

Statistical analyses for amino acids were performed using TIBCO Statistica^®^ 13.3 software. All data are reported as means (±SD) of 2 chemical determinations. Results were subjected to analysis of variance (ANOVA). Significant differences between means were determined by the Tukey test at *p* ≤ 0.05.

## 4. Conclusions

This study provided compositional and physicochemical characteristics of dried *Moringa oleifera* leaves based on amino acid analysis, antioxidant assay responses, chromatographic profiling, techno-functional measurements, and spectroscopic description.

The amino acid analysis demonstrated that *M. oleifera* leaves provide all essential amino acids, including tryptophan, which was determined using the validated procedure described in this study. The sulfur-containing amino acids and lysine were identified as the primary limiting factors, followed by histidine, leucine, and valine. Although the levels of some essential amino acids are lower than the reference pattern, the amino acid profile may be useful for comparative compositional evaluation of this material.

The solvent-dependent differences observed across antioxidant assays indicated method- and extraction-dependent variation in assay response. The ethanolic extract showed significantly higher reducing power and oxygen radical absorbance capacity, while radical scavenging activity and total phenolic content did not differ significantly between water and ethanolic extracts. These results should be interpreted as comparative characteristics of the tested extracts.

Chromatographic analysis confirmed the presence of fructose and glucose as the predominant sugars, along with several organic acids reflecting active primary metabolism and the diversity of soluble compounds in Moringa leaves. The techno-functional properties, including high water-holding capacity, notable water solubility, and considerable foaming capacity with good foam stability, describe the water-binding, solubility, and surface-active characteristics of the tested leaf powder. The material showed a predominantly hydrophilic character. The material’s predominantly hydrophilic nature further highlights its versatility in aqueous food systems.

Spectroscopic fingerprinting provided descriptive band patterns consistent with proteins, lipids, carbohydrates, and glycoside-related structures in the plant matrix. The preserved green-yellow coloration, supported by chlorophyll and carotenoid quantification, is consistent with retention of pigment-related features after processing.

In conclusion, this study expands the available compositional data for *Moringa oleifera* leaves and provides a revised analytical description of this material. The results should be interpreted within the scope of compositional and physicochemical characterization.

## Figures and Tables

**Table 1:** Profile and contents of amino acids (AA) and amino acid score (AAS) value of *M. oleifera* leaf meal (Mean ± SD).

Amino Acid	Amino Acid Contents		AAS * [%]
	g/100 g of DM	g/100 g of Protein	
Glu (glutamic acid)	1.612 ± 0.11 ^a^	9.99	
Asp (aspartic acid)	0.976 ± 0.07 ^b^	6.05	
Leu (leucine)	0.826 ± 0.06 ^bc^	5.12	83.93
Thr (threonine)	0.646 ± 0.05 ^cd^	4.01	160.40
Ala (alanine)	0.638 ± 0.04 ^cd^	3.95	
Arg (arginine)	0.629 ± 0.04 ^cd^	3.90	
Ile (isoleucine)	0.562 ± 0.04 ^cd^	3.49	116.33
Val (valine)	0.562 ± 0.03 ^cde^	3.48	87.00
Ser (serine)	0.509 ± 0.03 ^de^	3.16	
Phe (phenylalanine)	0.496 ± 0.04 ^def^	3.08	
Gly (glycine)	0.464 ± 0.04 ^def^	2.88	
Lys (lysine)	0.386 ± 0.12 ^defg^	2.39	49.79
Tyr (tyrosine)	0.321 ± 0.02 ^efg^	1.99	
Pro (proline)	0.264 ± 0.12 ^efg^	1.64	
Trp (tryptophan)	0.213 ± 0.00 ^fg^	1.32	200.00
His (histidine)	0.163 ± 0.01 ^g^	1.01	63.13
Cys (cysteine)	0.108 ± 0.02 ^g^	0.67	
Met (methionine)	0.075 ± 0.01 ^g^	0.47	
TOTALS	9.63	0.18	
Sulfur AA (Met + Cys)	0.183	1.14	49.57
Aromatic AA (Phe + Tyr)	0.817	5.07	123.66

SD—standard deviation; DM—dry matter; ^a–g^ means followed by the same letter are not statistically different; * values for AAS were calculated using the scoring patterns recommended by FAO/WHO [21]. The reference scoring pattern (g/100 g protein) for adults: His 1.6; Ile 3.0; Leu 6.1; Lys 4.8; sulfur AA 2.3; aromatic AA 4.1; Thr 2.5; Trp 0.66; Val 4.0.

**Table 2:** Retention time and concentration of organic acids and simple sugars in water and ethanolic extracts.

Retention Time, Min	Identified Compound	Concentration, mg/mL	
		Water Extract (WE)	Ethanol Extract (EE)
11.897	citric acid		0.192
11.828		0.673	
12.532	galacturonic acid	0.183	-
13.017	glucose		1.821
13.042		0.973	
13.703	fructose		2.569
13.738		3.024	
14.503	arabinose		0.166
14.828		0.255	
15.503	succinic acid		0.265
15.530		0.518	
16.625	lactic acid	1.275	-
18.737	acetic acid		0.136
18.715		0.737	

**Table 3:** Antioxidant activity, total phenolic content, and photosynthetic pigment profile of *Moringa oleifera* leaf extracts.

Extract	FRAP	DPPH	ABTS	ORAC	TPC	Ch-A	Ch-B	TC
	mgTE/g DM				mg GAE/g DM	mg/g DM		
H_2_O	24.93 ± 0.74 ^a^	14.04 ± 0.32 ^a^	2.67 ± 0.04 ^a^	96.68 ± 4.30 ^a^	19.71 ± 0.28 ^a^	-	-	-
80% E/M	27.05 ± 1.05 ^b^	13.47 ± 0.40 ^a^	2.59 ± 0.03 ^a^	107.24 ± 6.80 ^a^	19.41 ± 0.32 ^a^	-	-	-
	-	-	-	-	-	2.12 ± 0.02	0.37 ± 0.01	0.61 ± 0.01

DM—dry matter; Ch- A/B—chlorophyll a/b; different superscript letters within a column indicate statistically significant differences (Tukey’s test, *p* ≤ 0.05); 80% E/M—80% ethanol/methanol (*v/v*).

**Table 4:** Wavenumbers of the bands observed in the IR and Raman spectra of *M. oleifera* leaf samples and their proposed assignments [44,45,46,47,48,49,50].

FT-IR/ATR Peak Position (cm^−1^)	Raman Peak Position (cm^−1^)	Assignments
3288s		ν(O-H) (water, carbohydrates, flavonols)
	2930vs	ν_as_(CH_3_) (lipids)
2915vs	2908vs	ν_as_(CH_2_) (lipids)
	2885s	ν_s_(CH_3_) (lipids)
2849vs	2854m	ν_s_(CH_2_) (lipids)
1736m	1735w	ν(C=O) (esters, lipids, flavonoids)
1724sh		ν(C=O) (fatty acid esters)
1650sh	1651sh	ν(C=O) and δ(N-H) (amide I, proteins)
	1624sh	ν(C=C) (flavonoids)
1607m	1605s	ν(C=C) aromatic (flavonoids, phenolic compounds, e.g., rutin), δ(N-H) (amide I, overlap)
	1571m	ν(C=C) (flavonoids)
1550sh		δ(N-H) and ν(C-N) amide II (proteins), ν(C=C) (flavonoids)
1519m	1526m	ν(C=C) aromatic (flavonoids, lignins)
	1487m	δ(CH_2_) (lipids, proteins)
1472m		δas(CH_3_), δ(CH_2_) (lipids, proteins)
1463m	1462s	δ(CH_2_), δ(CH_3_) (lipids, proteins)
	1442w	
1411m	1412w	δ(O-H) (phenols, carboxylic acids), δ(CH_2_) (lipids)
1377m	1377m	δs(CH_3_) (proteins, lipids)
	1359m 1341m 1328m	δ(C-H) (flavonoids)
	1309m 1298m	ν(C-C) (flavonoids)
	1272m	ν(C-O) (phenols)
1238m	1239m	ν(P=O) (phospholipids), δ(C-O-H) (phenols)
1206sh	1206w	ν(C-O) (esters, phenols), δ(C-O-H) (flavonoids)
	1186w 1155m	ν(C-O) (carbohydrates)
1138sh		ν(C-O-C) (glycosides, carbohydrates)
	1125m	ν(C-O) (carbohydrates)
1097m	1093m	ν(C-O) (carbohydrates, secondary alcohols), ν(C-C), ν(P-O) (phospholipids)
	1060m	ν(C-O) (carbohydrates)
1046s	1041sh	ν(C-O) (carbohydrates, e.g., cellulose)
1024s		ν(C-O) (carbohydrates), ϕ (pyranoid ring)
	1003m	ν_s_(C-C) ring breathing (phenylalanine)
	958w	ϕ (pyranoid ring)
922w		ϕ (pyranoid ring), γ(C-H)
895w	895m	γ(C-H) (aromatics, carbohydrates)
	856m	γ(C-H) (aromatics)
834w		γ(C-H) (aromatics, isoprenoids)
	787m	γ(C-H) (aromatics)
770w		γ(C-H) (aromatics), ω(N-H) (proteins)
729w		γ(CH_2_), γ(C=C)/cis)
718w		γ(C-H) (alkyl side chains)
	690m	γ(C-H) (aromatics)
	618sh	δ(CCC) (aromatic rings)
	601m	δ(CCC) (aromatic rings)
594m		δ(C-C-C) (skeletal, ring deformations), δ(COO^−^)
	565sh 549w	δ(CCC) (rings)
533w	535m	δ(C-C-C) (skeletal, ring deformations), ω(COO^−^)
	520m 502m 461w 447w 434w 403sh 380m 331m 275w 261w 197m	e.g. δ(CCC) (rings)

s—strong; m—medium; w—weak; v—very; sh—shoulder; ϕ—pyranoid ring; ν—stretching; δ—in-plane bending vibrations; γ, ω—out-of plane bendig.

**Table 5:** Validation parameters for HPLC analysis of amino acids determined by acid hydrolysis.

Amino Acid	LOD [pmol/μL]	LOQ [pmol/μL]	RSD [%]	R^2^
Glu (glutamic acid)	8.84	26.79	2.82	0.99998
Asp (aspartic acid)	9.45	28.64	3.28	0.99998
Leu (leucine)	7.14	21.65	3.36	0.99999
Thr (threonine)	6.81	20.64	2.61	0.99995
Ala (alanine)	8.12	24.62	3.26	0.99992
Arg (arginine)	6.65	20.16	2.94	0.99992
Ile (isoleucine)	8.03	24.34	3.94	0.99999
Val (valine)	3.11	9.42	1.77	0.99998
Ser (serine)	6.60	20.00	2.56	0.99998
Phe (phenylalanine)	11.15	33.78	4.93	1.0000
Gly (glycine)	4.42	13.40	1.80	0.99992
Lys (lysine)	21.45	65.24	8.56	0.99954
Tyr (tyrosine)	10.30	31.20	4.25	0.99997
Pro (proline)	22.27	67.48	8.52	0.99971
His (histidine)	17.18	52.07	3.99	0.99988

LOD—limit of detection (n = 10), LOQ—limit of quantification (n = 10), RSD—repeatability (n = 6), R^2^—regression coefficient. LOD, LOQ, and RSD were estimated in accordance with ICH guidelines [62].

## Data Availability

The original contributions presented in this study are included in the article. Further inquiries can be directed to the corresponding author.
